# Soil zinc, serum zinc, and the potential for agronomic biofortification to reduce human zinc deficiency in Ethiopia

**DOI:** 10.1038/s41598-021-88304-6

**Published:** 2021-04-22

**Authors:** Hugo De Groote, Masresha Tessema, Samuel Gameda, Nilupa S. Gunaratna

**Affiliations:** 1 International Maize and Wheat Improvement Center (CIMMYT), PO Box 1041-00621, Nairobi, Kenya; 2 Ethiopian Public Health Institute, Gulelle Arbegnoch Street, Gulele Sub City, Addis Ababa, Ethiopia; 3 Wageningen University, Wageningen, The Netherlands; 4 Jimma University, Jimma, Ethiopia; 5 CIMMYT Ethiopia, P.O. Box 5689, Addis Ababa, Ethiopia; 6 Department of Public Health, Purdue University, 812 W State St., West Lafayette, IN 47907-2059 USA

**Keywords:** Plant sciences, Environmental sciences, Environmental social sciences, Health care

## Abstract

Human zinc deficiency is a global public health problem. Many African soils are zinc deficient (ZnD), indicating fertilizers could increase crop yields and grain Zn levels, thereby increasing Zn in the food supply and alleviating human Zn deficiency. To analyze associations among soil Zn, human Zn deficiency, and child nutritional status, we combined the Ethiopian soil Zn map and the Ethiopian National Micronutrient Survey (ENMS). ENMS provides representative, georeferenced data on child nutritional status using anthropometry of children under five years old (CU5) and on human Zn deficiency among CU5 and women of reproductive age (WRA) using the recommended biomarker, serum Zn. ZnD soils mostly occur in lower altitudes, which are less populated and outside the main crop-producing areas. Serum Zn deficiencies were high, and correlated to soil Zn for children, but not for WRA. About 4 million Ethiopian CU5 are ZnD, and, of these, about 1.5 million live on low-Zn soils (< 2.5 mg/kg), while 0.3 million live on ZnD soils (< 1.5 mg/kg). Therefore, if Zn fertilizers are only applied on ZnD soils, their impact on child Zn deficiency may be limited. Greater impact is possible if Zn fertilizers are applied to soils with sufficient Zn for plant growth and if this results in increased grain Zn. Optimal soil Zn levels for plant and human nutrition may be different, and context-specific optimal levels for the latter must be determined to develop nutrition-sensitive fertilizer policies and recommendations.

## Introduction

Undernutrition due to micronutrient deficiencies is prevalent in developing countries^1,2^. Human zinc deficiency, for example, is a global public health problem with important consequences for human health^3–5^. Mapping of soil fertility in Africa has shown zinc (Zn) deficiency to also be common in soils^6^, leading to the hypothesis that Zn-containing fertilizers could contribute to increased crop yields as well as increased grain Zn levels, which could then reduce human Zn deficiency by increasing the availability of Zn in the food supply^7^. This process is called agronomic biofortification^8^, in which crops can be “fortified” through agronomic means, and is similar to genetic biofortification^9^, in which the nutritional content of crops is increased through plant breeding. For agronomic biofortification to be successful, there must be a causal link between soil Zn and human Zn status in target populations, and Zn-deficient soils and Zn-deficient human populations must geographically coincide.

Zinc is a trace element essential for all life forms. It is involved in the production of a wide range of enzymes and proteins in plants, animals, and humans^10^. Recognition of its importance in human nutrition came relatively late^11^, but Zn deficiency is now considered an important public health issue, especially in developing countries^12^. It affects a range of health outcomes, including increased risk of mortality, stunting, diarrhea, and respiratory illnesses in children, preterm delivery in pregnant women, and immune function^13–15^.

Zinc deficiency in soils is an important constraint to crop production, and the most ubiquitous micronutrient deficiency in crops worldwide^10^, particularly limiting yields in developing countries^16,17^. Adding Zn to fertilizers is therefore a common strategy to enhance plant growth and increase yield^17^. Maize is the cereal most susceptible to Zn deficiency, although wheat and rice can also be highly prone^10^. In Ethiopia, a recent soil-fertility mapping exercise showed over 50% of agricultural soils to be Zn deficient, reaching over 70% in the Tigray region^18^. Diammonium phosphate (DAP) and urea have been the main fertilizers used in Ethiopia, supplying only two nutrients, phosphorus and nitrogen, while deficiencies in as many as eight nutrients, including Zn, were shown, leading to a national commitment to use blended fertilizers, i.e., fertilizers containing multiple nutrients, to address these deficiencies.

Human zinc requirements are relatively small, with 8 mg/day recommended for women and 11 mg/day recommended for men^19^. Still, an estimated 17.3% of the global population is at risk of insufficient Zn in their diet^20^, the main causes being insufficient intake or inadequate absorption^21,22^. Foods based on unrefined cereals and legumes tend to contain high levels of phytate, a potent inhibitor of Zn absorption^23^. In sub-Saharan Africa, where such diets are common, Zn deficiency is high^5,22^. Risk of Zn deficiency becomes a public health concern when the prevalence of inadequate Zn intake is higher than 25%^24^.

Ethiopia has particularly high rates of malnutrition and of adverse health outcomes associated with Zn deficiency: child mortality, morbidity, and stunting, and adverse perinatal outcomes in women^25–27^. Recent studies have quantified the burden of Zn deficiency in Ethiopia^3,28–31^. In 2011, 92% of children aged 1–3 years and 61% of adult women of reproductive age (WRA) had inadequate dietary intakes of Zn^32^.

Ideally, all people should have access to and consume a wide range of nutritious foods to fulfill their dietary needs; however, until that is achieved, complementary approaches are needed. Supplements and fortification of staple foods with micronutrients are common, but they reach the urban population more easily than the rural population, even though the latter are more affected^33^. Staple foods biofortified with micronutrients^34^ are an interesting alternative approach to reach rural populations, who have more limited access to diverse diets and nutritional interventions^9,35,36^. Genetic biofortification has resulted in increased provitamin A in orange-fleshed sweet potatoes^37^ and in orange maize^38^, and also led to high-iron beans^39^, and high-zinc and -iron durum wheat^40^.

For mineral micronutrients, agronomic biofortification can also help. In Finland, fortification of fertilizers with sodium selenite increased the selenium (Se) intake of the whole population, eliminating deficiencies^41,42^. Zinc fertilizer is used to increase yields on Zn deficient soils and has been particularly successful on wheat in Turkey^43^; it can also increase Zn in cereal grain^44,45^. Zinc fertilizer applied to maize has high potential for Africa^7^, and in particular for Ethiopia, where human Zn deficiency is high, soils are Zn-deficient, and maize has become the major food crop.

While agronomic biofortification can increase yields and increase micronutrient content of staple crops, this does not necessarily lead to improved human health^46^. The link between soil Zn and human Zn status, in particular, is not well understood. Recently, weak associations were found between soil nutrients, including Zn, and some health indicators, including child mortality and stunting in Sub-Saharan Africa^47^. In India, soil Zn affected Zn levels in rice and thus in the diet, but associations were not observed with serum Zn^48^. In Ethiopia, using the soil Zn map and the Ethiopian National Micronutrient Survey (ENMS), a significant correlation was found between soil Zn and serum Zn^49^. However, none of these observational studies provide strong evidence of causal relationships between soil Zn and human Zn status. Moreover, for agronomic biofortification with Zn to benefit human health, Zn deficient populations must live on Zn deficient soils, on which Zn fertilizer is applied and Zn in the harvested food supply is increased and consumed. It is not known whether these conditions could be met in Ethiopia or other countries that are potential targets for Zn agronomic biofortification (ZAB). Therefore, to help fill this gap, this research has the following objectives: (i) to map the geographic co-distribution of Zn deficiency in soils and in children under 5 years, a target population particularly vulnerable to Zn deficiency, (ii) to quantify the potential impact of ZAB by calculating the number of Zn-deficient children living on Zn-deficient soils in crop-growing areas, and (iii) to analyze the geographic distribution of that impact to inform nutrition-sensitive ZAB policies.

## Methods

### Conceptual framework and overview

**Figure 1:**
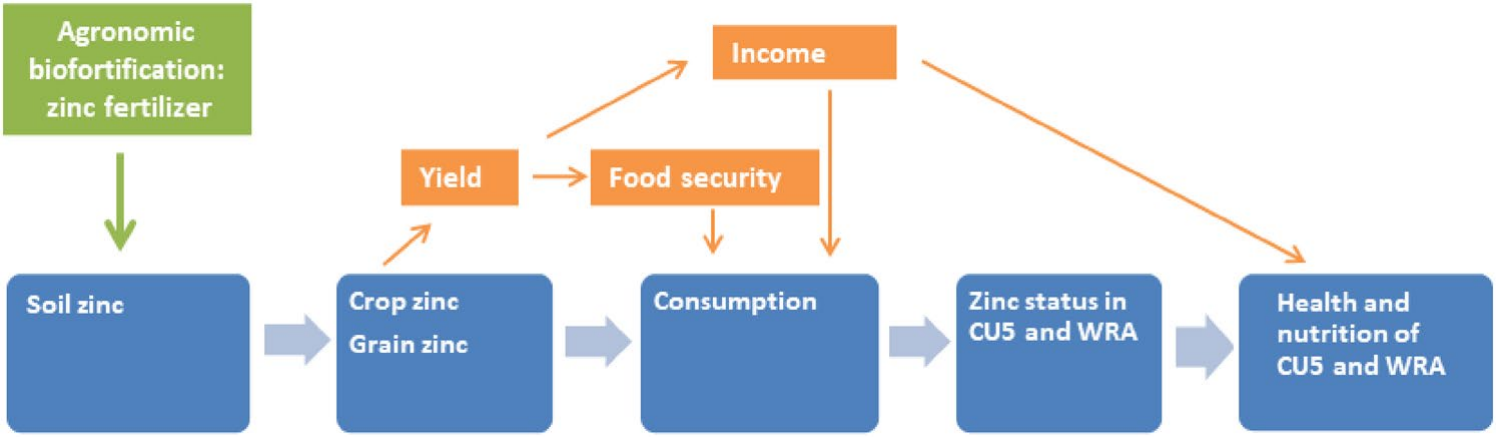
Conceptual framework of the potential impact of zinc agronomic biofortification of maize. (CU5: children under five years of age, WRA: women of reproductive age).

Conceptually, a direct link between low Zn in the soil and adverse health effects in humans can be hypothesized as follows (Fig. 1, direct effects in blue). Low soil Zn results in reduced plant growth and crop yields and low Zn levels in cereal grain^44^ and, when these comprise a substantial part of a diet low in alternative Zn sources, to low dietary Zn intake^48^ . This leads to poor Zn status, and infants, young children, and women of reproductive age are particularly vulnerable, with associated adverse effects on health outcomes^3,30,50^. Zinc agronomic biofortification increases both yields and grain Zn levels^44,51^, which could then result in higher overall dietary Zn intake, and improved zinc status in women and children (Fig. 1). Finally, higher Zn status can improve health outcomes. Increasing yields can also improve food security and/or income, indirectly improving health and nutrition outcomes (Fig. 1, indirect effects in orange).

To analyze these conceptualized relationships, we overlaid the soil zinc map from the African Soil Information System (AfSIS)^6^ with the Ethiopian National Micronutrient Survey (ENMS)^31^. Further, we combined these maps with the crop map and the population map to calculate the number of potential beneficiaries of ZAB on different soil types and regions of Ethiopia.

### Data sources and analysis

**Figure 2:**
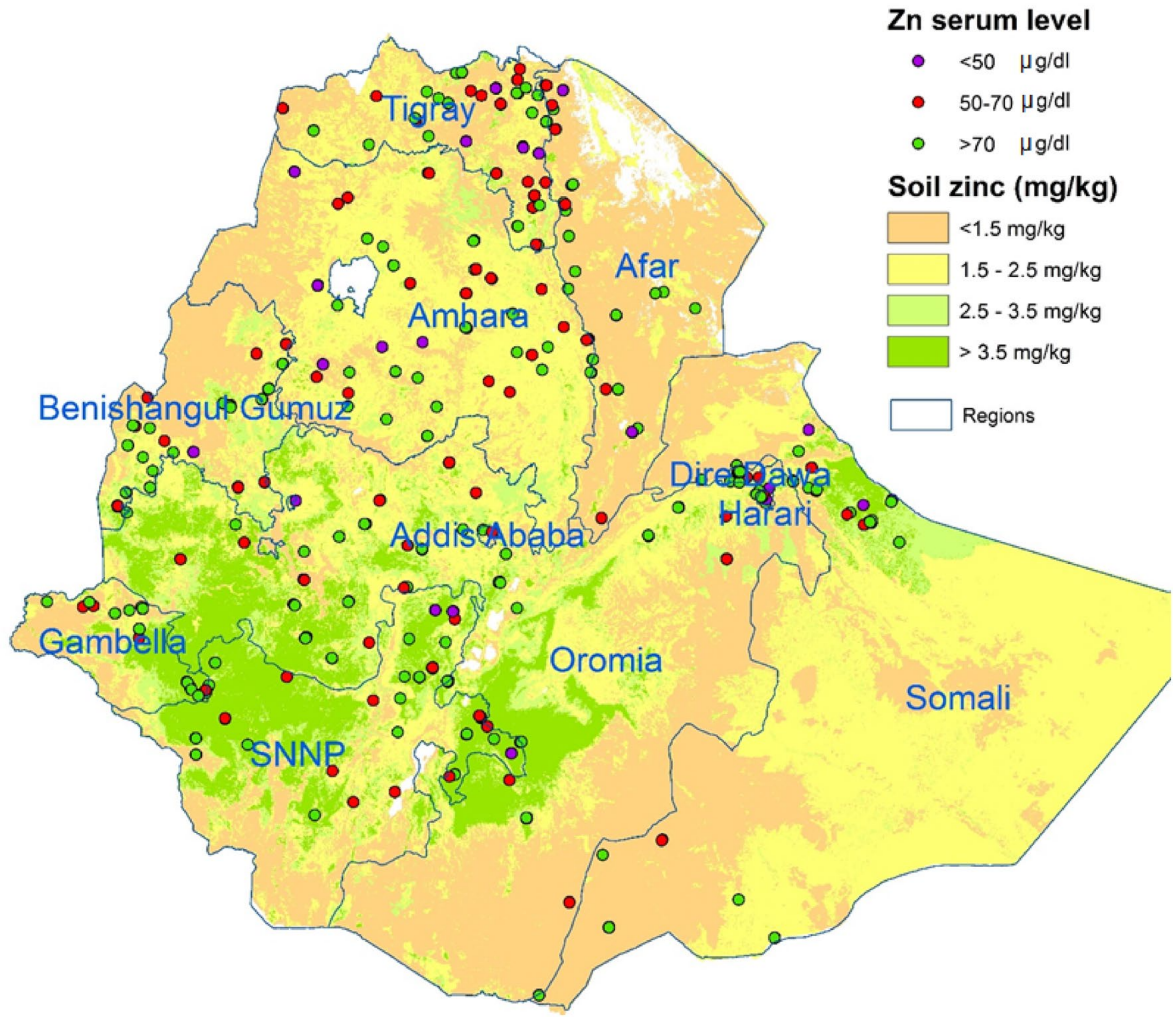
Map with soil zinc levels (AfSIS) and serum Zn levels in children under five years (ENMS) (map produced with ArcGIS Desktop version 10.8.1 from ESRI, https://www.esri.com/en-us/arcgis/products/arcgis-desktop/overview).

The soil zinc map of Ethiopia, based on systematic soil samples taken over most of Ethiopia, was obtained from the Africa Soil Information System (AfSIS, http://africasoils.net) (Fig. 2). This map represents extractable Zn levels as measured by the Mehlich 3 extraction method^52^. Zinc-deficient soils are defined as having extractable Zn levels lower than 1.5 mg/kg^53^, and soils with low Zn levels as those with levels between 1.5 and < 2.5 mg/kg. AfSIS also provided a crop mask: a grid layer indicating where crops were grown, developed with a machine-learning algorithm based on open source data^54^.

Data on Zn levels in human serum were obtained from the Ethiopia National Micronutrient Survey (ENMS), in which 3,805 households participated, randomly selected to be representative of the country’s regions. In these households, blood samples were collected from 1,776 children under five (CU5, aged between 6 and 59 months, of which 52% were girls), and 1,191 women of reproductive age (WRA, aged between 15 and 49 years old). From these, ENMS analyzed the serum Zn in 1,171 CU5 (Fig. 2) and 1,181 WRA.

The population of Ethiopia was obtained from the World Population layer (2015, http://www.worldpop.org.uk/), which is based on national census data and adjusted so that the totals reflect the national estimates. This data base has a layer of male and female CU5 (15.37% of the total), and a layer of WRA, estimated as those from 15 to 49 years old (24.81%).

### Analysis

As the ENMS data are representative at the regional level (the highest administrative level in Ethiopia), representative regional averages can be calculated and multiplied with regional populations to calculate the number of Zn-deficient people. As the ENMS data were georeferenced, the soil Zn levels for each data point could be extrapolated from the soil Zn map. The link between soil Zn and serum Zn was then analyzed, using serum Zn level and Zn deficiency as dependent variables and soil Zn level as an exploratory variable. Ordinary linear regression was used to analyze the effect of soil Zn (in mg/kg) on serum Zn (in µg/dL). Logistic regression was used to estimate the effect of soil Zn on the probability of a person being Zn-deficient, defined as serum Zn < 65 µg/dL for children (morning, non-fasting) and < 70 µg/dL for adult women (morning, fasting)^55^. The association was only significant for children, not for women, so only children were retained for further analysis.

Next, the serum Zn levels of children were extrapolated using Bayesian Kriging in ArcGIS Desktop (version 10.8.1 from ESRI, https://www.esri.com/en-us/arcgis/products/arcgis-desktop/overview) to produce a country-wide layer of the probability of Zn deficiency for children. This layer was then multiplied with the population layer to map the number of Zn-deficient children. By overlaying the resulting layer with the crop mask and the soil map, the number of Zn-deficient children living on agricultural soils, either Zn-deficient or with low Zn levels, could be calculated.

### Ethics approval

This study only uses secondary data: soil maps were obtained from AfSIS, and ENMS data were obtained from the Ethiopian Public Health Institute (EPHI), without identifiable variables such as names of the household members or participants. Ethical clearance for the ENMS was obtained from the National Research Ethical Review Committee of the Ethiopian Science and Technology Ministry (number 3.10/433/06), and informed consent was obtained from all adults who were interviewed, specifically the household head and caregiver, who also consented to the participation of their children. All methods were performed in accordance with the relevant guidelines and regulations.

## Results

### Population distribution

**Figure 3:**
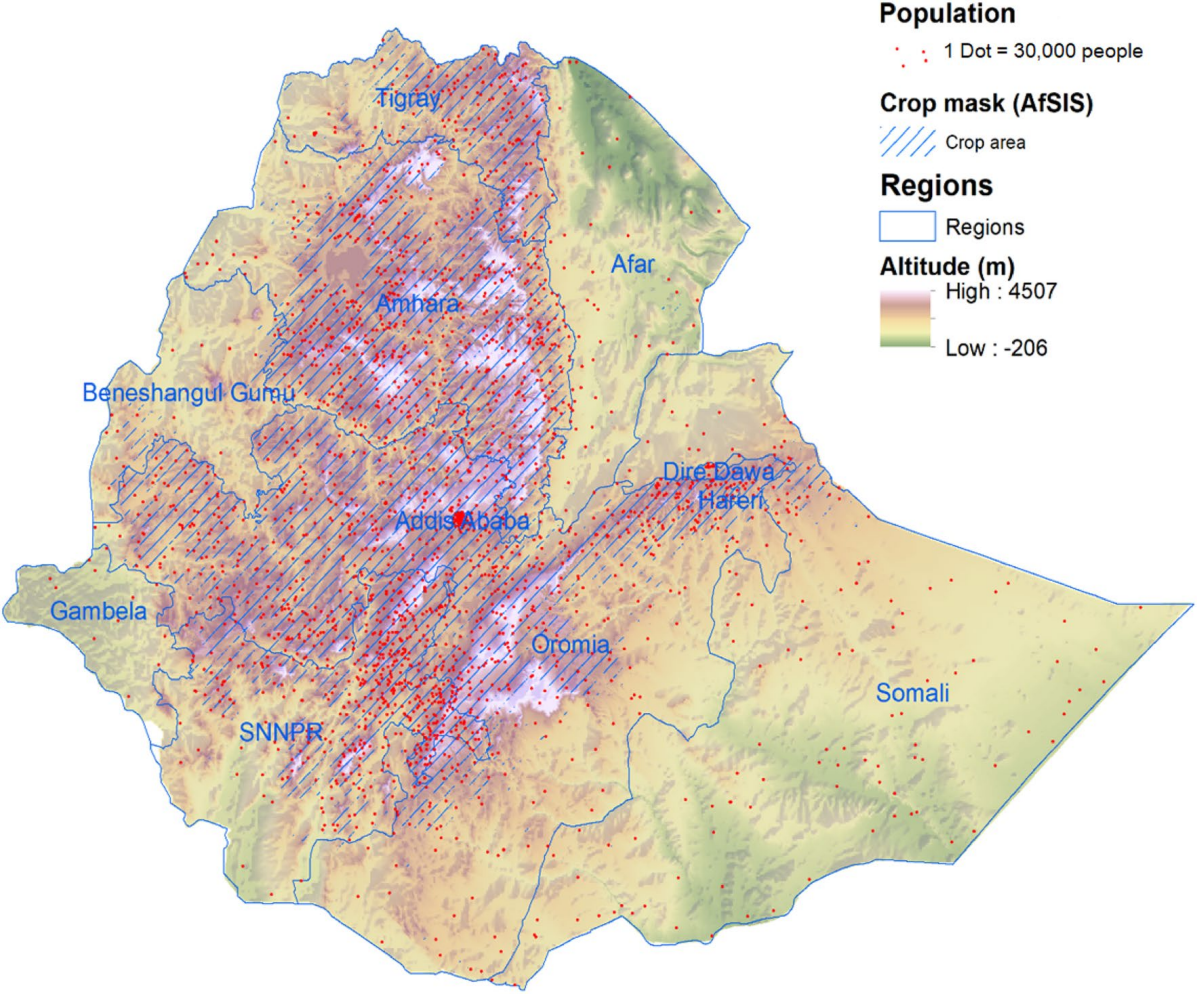
Population, altitude and crops in Ethiopia (map produced with ArcGIS Desktop version 10.8.1 from ESRI, https://www.esri.com/en-us/arcgis/products/arcgis-desktop/overview).

Before conducting our analysis of soil- and serum Zn, it was important to understand the population distribution in Ethiopia, as this would substantially affect the interpretation of the results. As shown in Fig. 3, the geography of Ethiopia is dominated by the Great Rift Valley, which transects the country from the South West to the North East, with highland areas on both sides. The SE highlands are narrow, located mostly in Oromia and partly in the Southern Nations, Nationalities and People’s region (SNNPR), while the NW highlands form a wide stretch and cover most of western Oromia, some parts of SNNPR, most of Amhara and a large part of Tigray. Almost all agricultural production takes place in these areas, and most of the population live there, including 9 million CU5 (out of 13.4 million).

### Analyzing the link between soil zinc and serum zinc

The soil map of Ethiopia shows that Zn-deficient soils (Zn < 1.5 mg/kg) are mostly found in the lower altitudes, located around, but not in, the highlands, which constitute the main agricultural areas in Ethiopia, except for some highland areas in Amhara and Tigray (Fig. 2). Low-Zn soils (1.5 < Zn < 2.5 mg/kg), on the other hand, cover a large part of northern and south-eastern Ethiopia, including most of Tigray and Amhara.

Children and women from the ENMS survey were defined as Zn-deficient when their serum Zn was below 65 and 70 µg/dL, respectively, and severely Zn-deficient if it was below 50 µg/dL. The average serum Zn level for children per cluster is presented in Fig. 2. The results show a high level of human Zn deficiency throughout the country, with weighted estimates of 28% of CU5 and 34% of WRA.

As the ENMS households were georeferenced, their position could be overlaid with the soil map and the interpolated soil Zn levels at these locations extracted. Analysis shows a significant positive correlation between serum Zn and soil Zn for children under five (Spearman’s ρ = 0.083, *p* = 0.006), but not for women of reproductive age. The results also show a significant correlation for children between soil Zn and weight-for-height z-score (WHZ) (ρ = 0.084, *p* = 0.001), but not between soil Zn and height-for-age z-score (HAZ), contrary to expectation.

Similarly, regression analysis shows a significant association of soil Zn with serum Zn in CU5, but not in WRA (Table 1). The linear regression shows that soil Zn levels have a small but significant association with children's serum Zn levels: for each mg/kg increase in soil Zn levels, serum Zn increases by 0.7 µg/dL. Similarly, soil Zn levels significantly reduce the probability that a child is Zn-deficient; with a coefficient (or log-odds ratio in this model) of -0.14, indicating that for each mg/kg increase of soil Zn, the odds of a child being Zn-deficient decrease by a factor of 0.87, or 13% (1–0.87). In other words, at the mean soil Zn level (2.7 mg/kg), an increase of one mg/kg leads to a decrease in child Zn deficiency of 2.8% (from 27.2 to 24.4%). The effect of soil Zn levels on women's serum Zn levels or on the probability of women being Zn-deficient was, however, not significant.

**Table 1:** Regression of serum zinc and zinc deficiency of children on soil zinc (all models estimated with random effects of the cluster variable, the 247 enumeration areas of the ENMS).

Group	Model	Dependent variable	Independent variables	Coefficient	Std. error	Sig
Children (N = 1038)	Linear model	Serum Zn (µg/dL)	Constant	74.994	1.044	< 0.001
			Soil Zn (mg/kg)	0.693	0.282	0.014
	Logistic model	Zn deficiency (serum Zn < 65 µg /dL)	Constant	− 0.668	0.143	< 0.001
			Soil Zn (mg/kg)	− 0.145	0.047	0.002
Women of reproductive age (N = 1191)	Linear model	Serum Zn, µg /dL	Constant	82.309	1.068	< 0.001
			Soil Zn (mg/kg)	− 0.267	0.320	0.405
	Logistic model	Zn deficiency (serum Zn < 70 µg /dL)	Constant	− 0.706	0.092	< 0.001
			Soil Zn (mg/kg)	0.021	0.027	0.448

**Figure 4:**
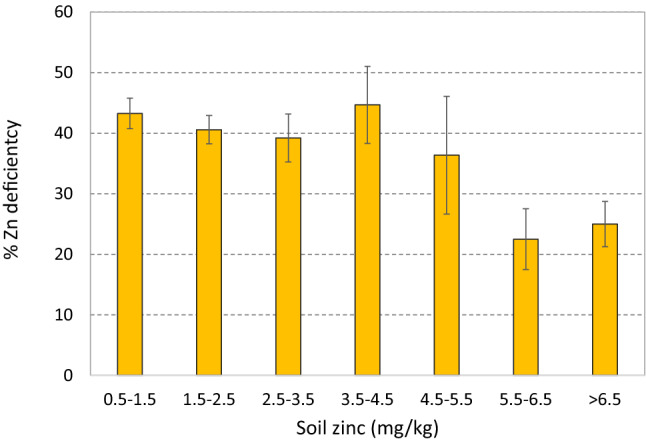
Zinc deficiency in children, by soil zinc level category (error bars are 95% confidence intervals).

Graphic analysis, however, indicates that the reduction of Zn deficiency in children due to soil Zn only kicks in at relatively high levels, around 4.5 mg/kg (Fig. 4). Therefore, children living on soils with Zn levels of 4.5 mg/kg or less are more likely to be Zn-deficient (on average more than 40%), while those on soils with Zn levels higher than 5.5 mg/kg or more are substantially less likely to be Zn-deficient (around 20%). For women of reproductive age, such a link is not clear. For children, on the other hand, the results indicate that there is potential for reducing Zn deficiency in children by agronomic biofortification of soils that had enough Zn for plant nutrition, but not for child nutrition. While the results do not allow calculation of a precise cut-off, the cut-off for human nutrition is likely substantially higher than that for plant nutrition.

### Calculating the number of potential beneficiaries

Based on the soil Zn levels from the AfSIS, the soils in Ethiopia can be divided into three categories: Zn-deficient (<1.5 mg/kg), low Zn (1.5–2.5 mg/kg) and sufficient Zn (>2.5 mg/kg) (Fig. 2). The average serum Zn levels of CU5, as obtained from the ENMS, were averaged over their clusters for clarity and added to the map. The map shows some higher levels of Zn deficiency in children on Zn-deficient and low-Zn soils, but not a strong link.

**Figure 5:**
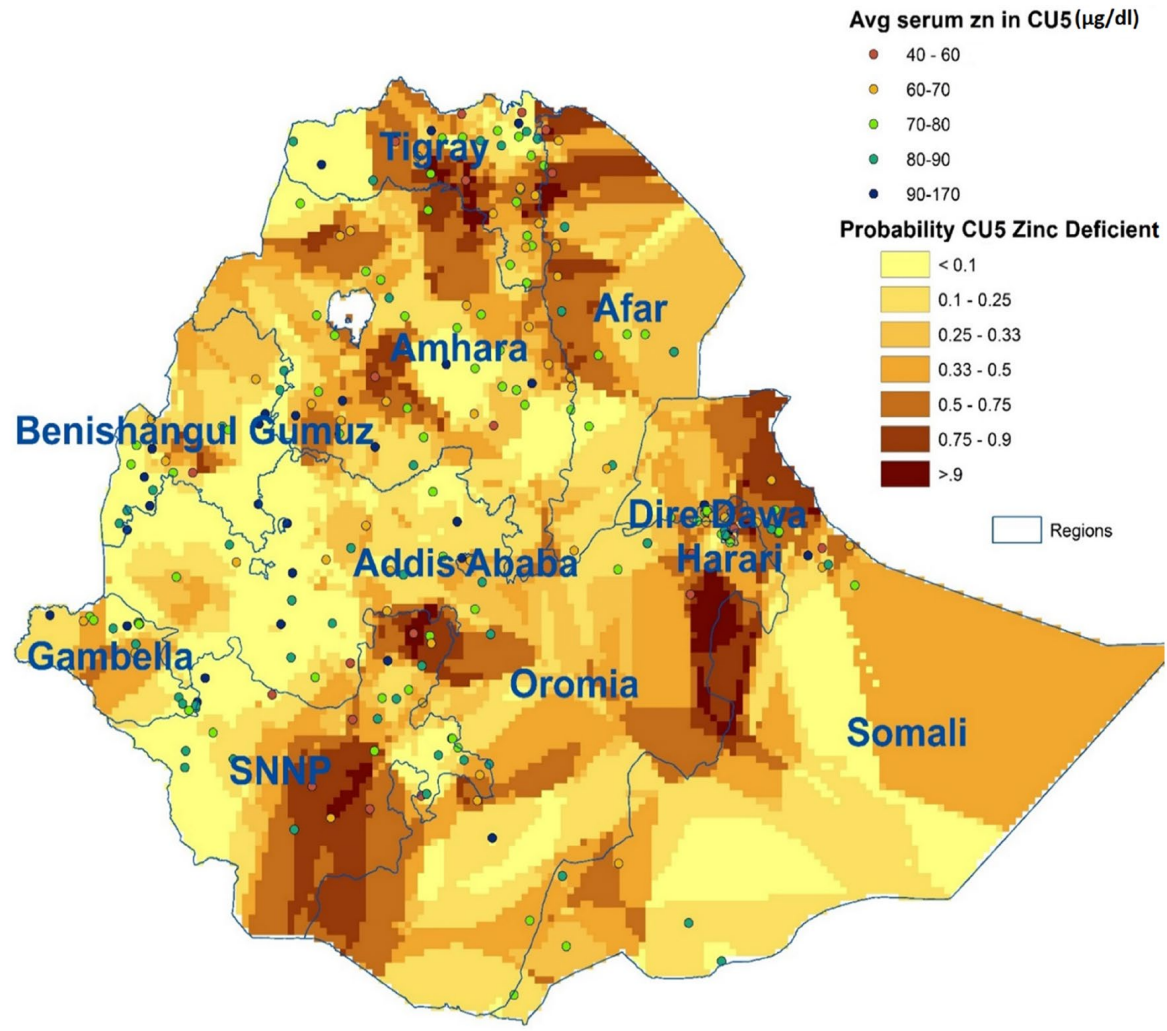
Average serum zinc in children under-five (per cluster, in µg/dL), and interpolated probability of zinc deficiency (serum zinc < 70 µg/dL) (map produced with ArcGIS Desktop version 10.8.1 from ESRI, https://www.esri.com/en-us/arcgis/products/arcgis-desktop/overview).

The individual serum Zn levels, georeferenced, were interpolated over the whole country using Bayesian kriging, resulting in a nation-wide geographic distribution of the probability of children under-five being Zn-deficient (Fig. 5). This map shows clear areas with a high probability of children being Zn-deficient, and these areas are more prevalent on Zn-deficient or low-Zn soils. Among agricultural areas, these include most of Tigray and large areas of Amhara and SNNPR, but not Oromia.

**Figure 6:**
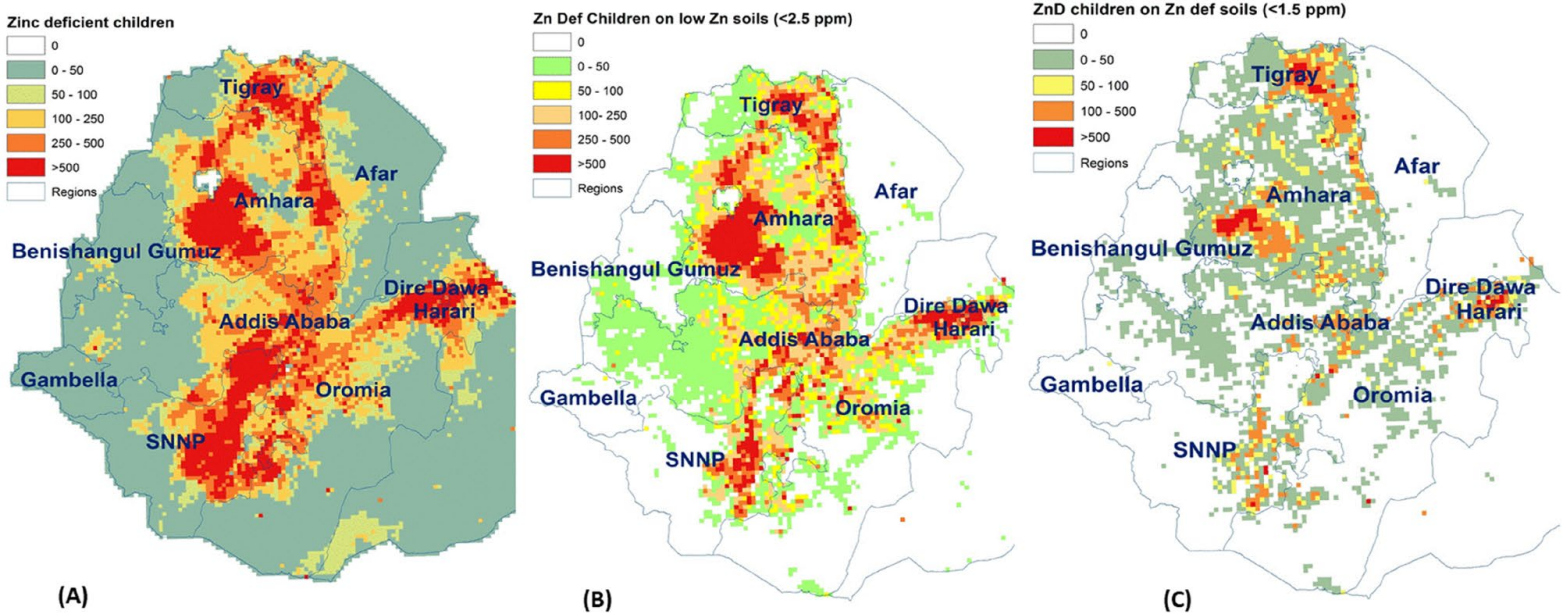
Mapping the number of zinc-deficient children, by zinc soil type (map produced with ArcGIS Desktop version 10.8.1 from ESRI, https://www.esri.com/en-us/arcgis/products/arcgis-desktop/overview).

Next, the probability of Zn deficiency among children was multiplied by the actual number of children under five years of age (0–59 months) in each cell, resulting in a map of the number of Zn-deficient children (Fig. 6, Panel A). The results show high numbers of Zn-deficient children in most agricultural and high population-density areas. When we calculate the number of Zn-deficient children on low-Zn soils (Fig. 6, Panel B), clear areas of concern emerge, in particular most of Tigray and large parts of Amhara, as well as an axis along the Rift Valley from Dire Dawa, over Oromia, to SNNPR. However, if we limit the analysis to Zn-deficient children on Zn-deficient soils (Fig. 6, Panel C), the areas of interest are much reduced, and mostly limited to five distinct geographical areas: eastern Tigray, southern Amhara, central and northeastern Oromia and southeastern SNNPR. The main reason for the reduction is the lower population density in areas with Zn-deficient soils.

The data from these maps were then aggregated to calculate the number of Zn-deficient children on the three soil categories, and for each region (Table 2). The results show that out of the 13 million Ethiopian CU5, about five million (37%) live on low-Zn soils, and less than one million (7%) on Zn-deficient soils. The regions with the highest number of children on Zn-deficient soils are Amhara (345,000), Oromia (238,000) and Tigray (almost 200,000).

**Table 2:** Number of children under five years of age, total and zinc deficient, living on zinc deficient and low zinc soils, by region.

	Children under five			Zinc deficient children under five							
	All	On low Zn soils with crops		All, from ENMS				All, from extrapolation		On low Zn soils with crops	
		< 2.5 mg/kg	< 1.5 mg/kg					< 2.5 mg/kg		< 1.5 mg/kg	
				N	%	N	%	N	%	N	%
Tigray	780,757	407,489	197,379	281,073	36	291,799	37	147,974	36	75,722	38
Afar	255,676	5572	4886	102,270	40	94,332	37	1371	25	998	20
Amhara	3,412,420	2,173,250	345,247	1,023,726	30	1,124,970	33	721,003	33	119,625	35
Benishangul Gumuz	123,717	29,137	6359	24,743	20	14,897	12	7145	25	1452	23
Oromia	4,694,730	1,639,390	238,066	1,173,683	25	1,213,720	26	388,088	24	64,104	27
Somali	754,297	26,084	7748	181,031	24	276,460	37	8425	32	2107	27
Dire Dawa	73,982	5,209	1795	21,455	29	35,921	49	1956	38	1091	61
Harari	36,500	14,548	8225	11,680	32	3475	10	3870	27	2624	32
Addis Ababa	587,807	61,555	10,014	352,684	60	204,369	35	13,426	22	2388	24
SNNPR	2,618,460	546,970	72,000	759,353	29	1,045,530	40	198,904	36	32,858	46
Gambella	44,657	4,296	4230	5805	13	9290	21	1009	23	880	21
	13,383,003	4,913,499	895,950	3,937,504	28	4,314,763	32	1,493,172	30	303,849	34

The ENMS averages, by region, find the highest levels of Zn deficiency among children under five in Afar (40%), Tigray (36%), Addis Ababa (60%) and Harari (32%), with a national average of 28%. As these results are representative for the regions, these averages can be multiplied by the number of CU5. The results show that the largest number of Zn-deficient under-fives are found in Oromia (1.2 million), Amhara (1.0 million), and SNNPR (0.8 million). Similar results were obtained by multiplying the grid with the probability of Zn deficiency, with the grid with the number of under-fives, albeit with a slightly higher estimate of the total proportion of deficient children (32%).

Using the grid levels instead of regional averages, however, has the advantage that the number of Zn-deficient children can now be calculated per soil type. The results show almost 1.5 million Zn-deficient children on low-Zn soils, mostly in Amhara (0.72 million), Oromia (0.39 M), SNNPR (0.20 M) and Tigray (0.15 M). On Zn-deficient soils, on the other hand, we only find a low number of Zn-deficient children, 0.3 M in total, most of whom are in Amhara (120 k), Tigray (76 k), Oromia (64 k) and SNNPR (33 k).

## Discussion

This paper set out to explore the link between soil zinc deficiency and human zinc deficiency using two nationally representative data sets, the Ethiopian soil Zn map and the ENMS, and to quantify the potential for Zn fertilizers to improve human Zn deficiency. The soil map shows a clear pattern of Zn-deficient soils located in the lower altitudes of Ethiopia, which have lower population density and less crop production. The lack of coincidence of Zn deficient soils, high population, and high levels of crop production raises the question of whether ZAB can have significant impact on human nutrition or health in Ethiopia. Meanwhile, the ENMS data show high levels of Zn deficiency among children and women, but without clear geographic patterns.

Combining the two data sets produces two main results. First, statistical analysis shows a significant, albeit small, association between soil Zn and serum Zn and Zn deficiency in children, but not in women. While this relationship is based on observational data and does not provide causal evidence, it does motivate further examination of the potential for ZAB to improve the nutritional status and health of young children. However, our calculations indicate that only about 300,000 Zn-deficient CU5 live on Zn-deficient soils in agricultural areas. If Zn fertilizers are only applied to Zn-deficient soils, i.e., soils with inadequate Zn for crop growth, any potential impact would be limited to those children, and large investments in soil Zn fertilizers to improve human health may not be justified. Further agronomic research is needed to establish whether Zn fertilizer applied to soils with higher Zn levels will still increase grain Zn levels for the benefit of consumers. If so, Zn-deficient children who live on more fertile soils may benefit. Our calculations show, for example, that if Zn fertilizer could increase Zn grain levels not just on Zn-deficient soils (< 1.5 mg/kg) but also on low-Zn soils (from 1.5 to 2.5 mg/kg), almost 1.5 million Zn-deficient children could be reached. However, even if Zn fertilizers are effective in increasing grain Zn on non-Zn deficient soils^51^, significant changes in agricultural policies and practices would be needed before Zn fertilizers are used only for biofortification, without an agronomic need or yield benefit, and it is not clear who would shoulder the associated additional costs.

The second main result is that significant reduction in the prevalence of Zn deficiency in young children was only observed at high soil Zn levels. Even with a causal effect of soil Zn on children’s Zn status, a key question remains on whether the amount of Zn fertilizer applied would result in an increase in soil fertility adequate to ultimately achieve reduced risk of human Zn deficiency. This finding suggests that the minimum adequate soil zinc level for human nutrition could be substantially higher than that for plants. Unfortunately, our data are too limited to calculate this minimum, and this value will likely vary by target population and context, depending on the severity of human zinc deficiency, other available dietary sources of Zn, soil properties that affect crops’ Zn uptake, and other factors.

While Zn deficiency is an important public health problem, assessment of Zn status poses technical challenges^55,56^. Zinc in serum or plasma, despite its limitations, is the recommended biomarker, and linear growth (height-for-age) in CU5 is the only recommended functional indicator, hence the usage of these measures in this paper. Our estimates of Zn deficiency based on serum Zn are much lower than those based on food consumption^25,26^. As the Ethiopian soil map is available online and in the public domain, the present study can easily be expanded to other biomarkers or other indicators of Zn deficiency that are georeferenced. However, such data are not likely to be widely available and representative of a target population, complicating analysis of the wider potential nutritional and health effects of ZAB across the life course.

An alternative or complementary option to soil Zn fertilizers would be foliar Zn applications. These have only a small or no effect on yields on Zn deficient soils, as compared to soil Zn applications; however, foliar Zn applications increase grain Zn across a range of soil Zn levels and do so more efficiently than soil Zn applications^43,51,57^. This efficiency varies by crop and soil type, which in turn vary by altitude. On wheat, grown in the higher altitudes of Ethiopia, soil Zn applications on Zn deficient soils increase grain yields, but foliar applications do not^43,57^. Similar results were found on peas^58^. Meanwhile, foliar applications on maize (usually grown at altitudes lower than for wheat) and barley (grown at higher altitudes) did increase yields^59,60^. In maize, a small yield effect was found from foliar spray in pot trials, but a larger effect from soil application, and the highest with the combination^60^; for grain Zn, on the other hand, foliar application had a stronger effect than soil application, and again the highest levels were obtained with a combination. In barley, a positive effect of foliar spray on both yield and grain Zn was found^59^. Rice, a low-land crop, appears to respond similar to wheat: Zn fertilizer had little effect on rice grain yield, but increased grain Zn^61^.

On the positive side, as zinc fertilizer can increase grain zinc in both wheat^51^ and maize^62^ on soils that are not zinc-deficient, the impact of zinc fertilizer on human health can go beyond its use on zinc-deficient soils. We do not know at this stage how far, but the Geonutrition Project^63^ is conducting studies aiming to find the critical level of soil zinc to meet human nutrition needs. As we show here, if zinc fertilizer is effective on low-zinc soils, one and a half million zinc-deficient children could be reached. It should be further noted that our cutoff to indicate low soil zinc (2.5 mg/kg) is arbitrary, as currently we do not have an empirical basis for such a limit. On the negative side, this would mean that zinc fertilizer on low-zinc soils might not increase crop yields. With no increase in yield, a farmer would not benefit from use of zinc fertilizer, and the extra cost of that fertilizer would therefore need to be covered by subsidies. The farmer could benefit if there were a premium for biofortified grain; however, this is unlikely as grain biofortified with zinc cannot be easily distinguished from non-biofortified grain, and in this scenario, any benefit would be monetary rather than nutritional for the farming household. Hence, the cost effectiveness of agronomic biofortification needs to be compared to other interventions such as genetic biofortification, as has been argued before^46^. As the effects of soil nutrients on human health in Africa have been found to be fairly small, agronomic biofortification might not be the most cost-effective way to reduce malnutrition, except for a few regions^47^.

The fertilizer blending initiative in Ethiopia provides a good opportunity to add Zn to soil fertilizer, which would increase crop yield on Zn deficient soils, but would only have a small effect on grain Zn, and only on those soils. As our results show the relatively lower cultivation of crops on Zn deficient soils, the impact could be small. As we describe above, foliar sprays are an alternative or complementary strategy to increase grain Zn, even on soils that are not Zn deficient, but they would have little or no effect on grain yields^43,51^. Therefore, foliar sprays should be considered in an overall biofortification strategy, in combination with the best crop varieties^43^. However, unlike soil Zn application, in which Zn can just be added in a fertilizer blending facility, foliar spray requires a very different approach and the involvement of farmers in application. To find the optimal strategy, more experiments are needed across crops and target environments, along with careful economic analysis.

For an integrated strategy, both genetic and agronomic fortification will probably be needed^43,64,65^. Genetic biofortification is sustainable and cost-effective to increase grain Zn^36,44^. While genetic variation of Zn concentration is lower in grain than in leaves, it still has sufficient variation for breeding^66^. However, it is a long-term process requiring substantial efforts and resources; while foliar or combined soil and foliar application of Zn fertilizers offer highly effective and practical short-term solutions^44^. Moreover, while this will help with micronutrient deficiencies, other measures are needed to solve the wider food insecurity problem in Ethiopia^67^.

In conclusion, our study confirms that Zn deficiency is a serious public health problem in Ethiopia, as has been shown in other countries^30^, and that it is related to low soil Zn, as we had shown earlier^49^. We expanded that analysis by calculating the potential impact in terms of the number of Zn-deficient children that could be reached. This strengthens the evidence on the potential of ZAB^7^. However, we also show that, in Ethiopia, the main agricultural soils are not Zn-deficient, and as the human population is concentrated on those soils, the potential impact of Zn fertilizer on Zn-deficient soils, at least by soil application, is limited. However, if grain Zn could be increased through Zn fertilizer on less deficient soils, through soil or especially foliar application, the potential could be much larger, depending on the maximum soil Zn level up to which Zn fertilizer is effective in increasing grain Zn. More experimental work is needed to determine that maximum level, to calculate the total area for which Zn fertilizer would be effective, and to calculate the associated cost of Zn agronomic biofortification through both soil and foliar applications. From there, the potential impact in terms of the number of Zn-deficient children that could be reached can be calculated.
